# Incidence of cardiovascular disease and its associated risk factors in at-risk men and women in the United Arab Emirates: a 9-year retrospective cohort study

**DOI:** 10.1186/s12872-019-1131-2

**Published:** 2019-06-17

**Authors:** Saif Al-Shamsi, Dybesh Regmi, Romona D. Govender

**Affiliations:** 1 Department of Internal Medicine, College of Medicine and Health Sciences, United Arab Emirates University, Al Ain, United Arab Emirates; 2 Department of Family Medicine, College of Medicine and Health Sciences, United Arab Emirates University, Al Ain, United Arab Emirates

**Keywords:** Cardiovascular disease, Myocardial infarction, Stroke, Acute peripheral arterial occlusion, Risk factors, Incidence, United Arab Emirates

## Abstract

**Background:**

Cardiovascular disease (CVD) is the leading cause of mortality worldwide; however, the epidemiology of CVD among nationals from the United Arab Emirates (UAE) remains unknown. This study aimed to estimate the 9-year incidence rate of CVD and determine the risk factors associated with CVD among UAE nationals at high cardiovascular risk. In addition, we investigated whether sex was an important modifier of the risk factors associated with incident CVD in this population.

**Methods:**

A retrospective cohort study was conducted on 977 subjects, including 492 women, aged ≥18 years, who did not have histories of CVD, and who had ≥1 CVD risk factors. Multivariable Cox proportional hazards regression analyses stratified by sex were used to examine the predictors of major CVD events, namely, myocardial infarction (MI), stroke, and acute peripheral arterial occlusion.

**Results:**

During a median follow-up period of 8.9 years, the incidence rate of major CVD was 12.7 per 1000 person-years (95% confidence interval [CI] 10.4–15.4), and among men and women were 16.8 (95% CI 12.9–21.4) and 9.0 (95% CI 6.4–12.4) per 1000 person-years, respectively. Major CVD and MI were significantly more frequent among men than women, and the stroke and acute peripheral arterial occlusion rates were similar for both sexes. Multivariable Cox analyses showed that the systolic blood pressure, estimated glomerular filtration rate, and serum glycosylated hemoglobin A1c level were strong predictors of major CVD in both sexes. Among women, the total cholesterol (TC)-to-high-density lipoprotein-cholesterol (HDL-C) ratio (hazard ratio [HR] 1.44, 95% CI 1.02–2.04) was an additional independent predictor of major CVD. Age (HR 1.50, 95% CI 1.19–1.89) and a history of smoking (HR 1.80, 95% CI 1.07–3.02) were significant risk factors associated with major CVD in men.

**Conclusions:**

Among high-risk UAE nationals who did not have histories of CVD, the risk of major CVD was associated with high systolic blood pressure, a low estimated glomerular filtration rate, and poorly controlled diabetes. The high TC-to-HDL-C ratios, especially among women, and smoking among men, are modifiable risk factors that should be managed aggressively.

**Electronic supplementary material:**

The online version of this article (10.1186/s12872-019-1131-2) contains supplementary material, which is available to authorized users.

## Background

Cardiovascular disease (CVD) is the leading cause of death worldwide, and it will continue to dominate future trends in global mortality [1]. In 2016, approximately one-third of all deaths worldwide were caused by CVD [2]. Ischemic heart disease was the principal cause of death associated with CVD, followed by stroke [3].

Since the 1970s, the United Arab Emirates (UAE) has undergone a tremendous transformation from an impoverished region to an affluent modern state with a high standard of living. This has resulted in drastic lifestyle changes among the inhabitants of the UAE, and, consequently, increases in the risk factors associated with non-communicable diseases, including CVD [4, 5].

Older age, obesity, hypertension (HTN), dyslipidemia, smoking, type 2 diabetes (DM), being male, and chronic kidney disease (CKD) are well-known risk factors associated with CVD [6].

Furthermore, combinations of these risk factors exert unfavorable synergistic effects on long-term survival [7–10]. The UAE has a higher prevalence of CVD risk factors than developed countries [5], and the prevalence of deaths associated with CVD among UAE nationals is higher than the global average [11, 12]. Thus, CVD is the main health threat in the UAE.

Although the risk factors for CVD have been identified within the general population [5], similar data for UAE nationals at high cardiovascular risk are lacking. Furthermore, the incidence rate of CVD in this high-risk population remains unknown. Gaining an understanding of the epidemiology of CVD is necessary to better inform current and future actions, including decisions related to policy making for high-risk UAE nationals. This study aimed to estimate the incidence rate of CVD and to determine the risk factors associated with CVD among UAE nationals with ≥1 CVD risk factors. In addition, we investigated whether sex was an important modifier of the predictors of incident CVD among high-risk UAE nationals.

## Methods

### Study setting

This retrospective study reviewed ambulatory electronic medical records of patients who presented to outpatient clinics at Tawam Hospital, Al-Ain, between April 1, 2008, and December 31, 2008. Al-Ain is the fourth largest city in the UAE, and it has a population of approximately 200,000 UAE nationals [12]. Tawam Hospital is a publicly funded facility, and, collectively, its outpatient medical clinics serve most of the residents of Al-Ain. Ethical approval for this study was obtained from Tawam Hospital and the United Arab Emirates University’s Research and Ethics Board (CRD 239/13). The requirement for informed consent was waived because the patients’ records and information were anonymized and de-identified before the analyses.

### Subjects and procedures

The study’s sample size was calculated using a formula for a study designed to estimate incidence in a population [13]. A sample size of 923 was determined based on an anticipated 10% incidence of CVD [6] and utilizing 80% power at a 2-sided level of significance of 0.05.

Sociodemographic and clinical data were collected through a systematic review of 1534 consecutive patients’ ambulatory electronic medical records. The study’s inclusion criteria were UAE nationals aged ≥18 years who had any of the following at baseline: a history of smoking, body mass index (BMI) ≥25 kg/m^2^, systolic blood pressure (SBP) ≥120 mmHg, diastolic blood pressure (DBP) ≥80 mmHg, serum glycosylated hemoglobin A1c (HbA1c) level ≥ 5.7%, serum triglyceride (TG) level ≥ 1.69 mmol/L, serum total cholesterol (TC) level ≥ 5.17 mmol/L, serum low-density lipoprotein-cholesterol (LDL-C) level ≥ 3.36 mmol/L, and a serum high-density lipoprotein-cholesterol (HDL-C) level < 1.03 mmol/L, and lipid-lowering, antidiabetic, or antihypertensive medications.

**Fig. 1:**
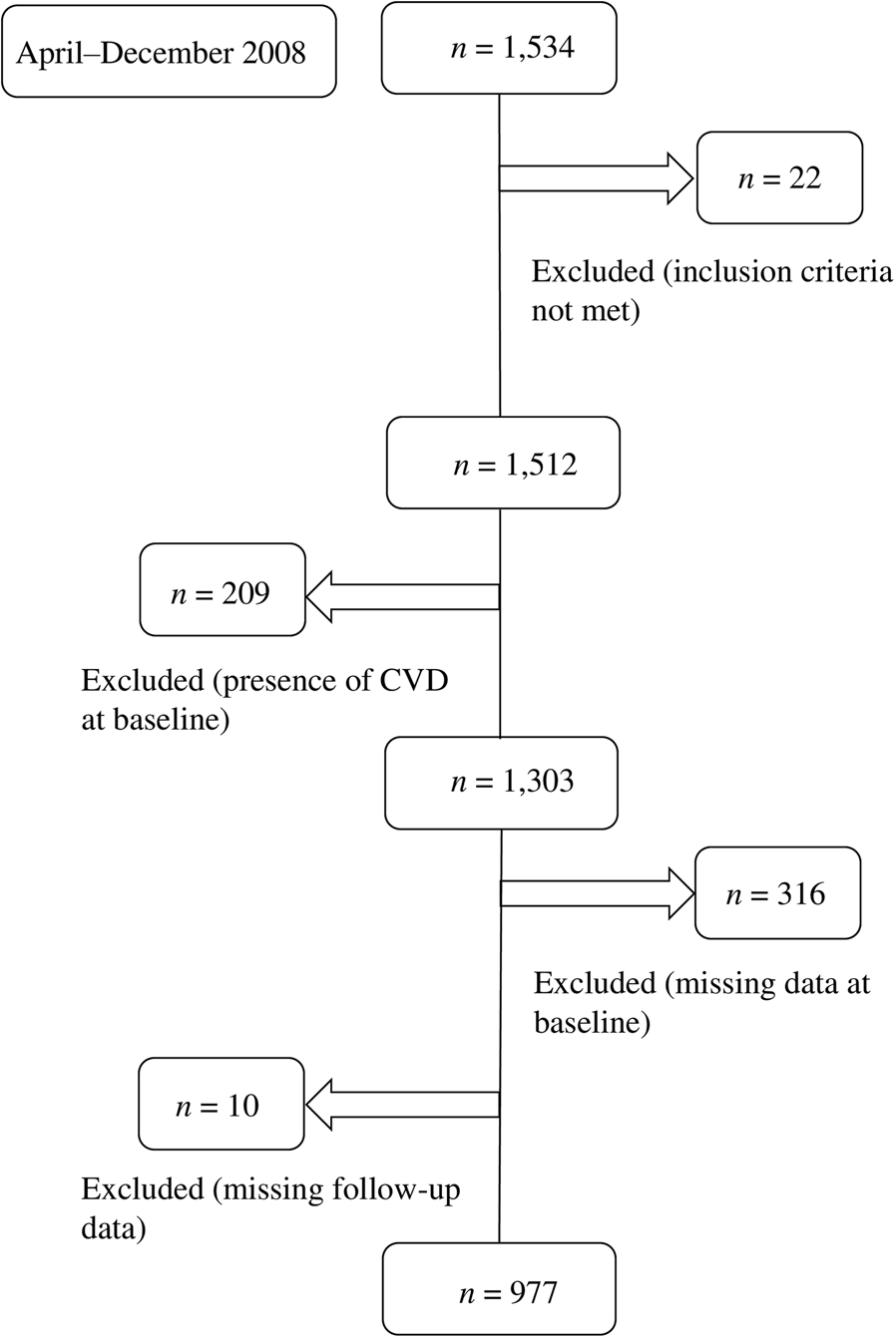
Subject selection flowchart. *CVD* cardiovascular disease

Of the 1512 eligible subjects, we excluded 209 subjects who had histories of a CVD event, which was defined as a diagnosis of angina, a prior myocardial infarction (MI), angioplasty of the coronary arteries, coronary artery surgery, a stroke, a transient ischemic attack, peripheral arterial disease, or heart failure, at baseline. We also excluded 316 subjects whose data were missing at baseline. Follow-up data were collected annually between the baseline visits in 2008 and July 31, 2018. During the follow-up period, ten subjects, representing 0.77% of the initial cohort, had < 1-year follow-up after inclusion. These subjects were excluded from the final analyses. The final sample for the analyses comprised of 977 subjects (Fig. 1).

### Measurements and definitions

The data collected at baseline included sociodemographic parameters, clinical information, and treatment modalities. The sociodemographic data included age, sex, and history of smoking, which was defined as a current or past history of smoking tobacco. The clinical data included the BMI, which was calculated as the weight (kg) divided by the height (m^2^), SBP and DBP, the HbA1c, TC, HDL-C, LDL-C, and TG levels, the TC/HDL-C ratio, and the estimated glomerular filtration rate (eGFR). The eGFR was calculated using the Chronic Kidney Disease Epidemiology Collaboration equation that is based on serum creatinine [14]. The treatment modalities included the use of antidiabetic medications, lipid-lowering agents, and antihypertensive drugs. Dyslipidemia was defined as documented treatment with lipid-lowering medications (e.g., bile acid sequestrant, fibrate, or 3-hydroxy-3-methylglutaryl coenzyme A reductase inhibitor) or the presence of ≥1 of the following: TG ≥1.69 mmol/L, TC ≥5.17 mmol/L, LDL-C ≥ 3.36 mmol/L, or HDL-C < 1.03 mmol/L [15]. Obesity was defined as a BMI of ≥30 kg/m^2^. Blood pressure (BP) was measured by qualified registered nurses using an automated oscillometric BP measuring device as part of the daily routine clinical practice. HTN was defined as an SBP ≥140 mmHg, a DBP ≥90 mmHg, or the use of antihypertensive medications (e.g., diuretics, angiotensin-converting enzyme inhibitors, angiotensin receptor blockers, alpha blockers, beta blockers, or calcium channel blockers). According to the Seventh Report of the Joint National Committee on Prevention, Detection, Evaluation, and Treatment of High Blood Pressure [16], we categorized BP into three groups: Those with SBP lower than 139 mmHg and DBP lower than 89 mmHg as normal BP group; Stage 1 HTN was defined as an SBP between 140 and 159 mmHg, or DBP between 90 and 99 mmHg; Stage 2 HTN was defined as an SBP higher than 160 mmHg or DBP higher than 100 mmHg. If systolic and diastolic pressures fell into different categories, subjects were assigned to the higher category. Patients with an HbA1c level of ≥6.5% or who were receiving antidiabetic medications (e.g., sulfonylurea, thiazolidinedione, dipeptidyl peptidase-4 inhibitor, biguanide, or insulin) were considered to have DM. Good glycemic control was considered if patients with DM had a HbA1c level of < 7% [17]. CKD stages 3–5 were defined as an eGFR < 60 mL/min/1.73 m^2^ according to the Kidney Disease: Improving Global Outcomes clinical guidelines [18].

### Outcomes

A major CVD event was defined as the first occurrence of a fatal or non-fatal MI, a fatal or non-fatal stroke, or acute peripheral arterial occlusion. A non-fatal MI was confirmed using the World Health Organization’s criteria in the context of elevated levels of cardiac biomarkers with either symptoms or diagnostic electrocardiograms suggestive of ischemia [19]. A non-fatal stroke was defined as typical neurological dysfunction lasting > 24 h and neuroimaging that excluded other diagnoses. The occurrence of fatal MIs or fatal strokes was confirmed by the hospital records or death certificates. Sudden death outside the hospital after the onset of symptoms with no evidence of a non-coronary cause of death was also considered a fatal MI. Acute peripheral arterial occlusion was defined as an arterial event that had a sudden onset, had a duration of < 2 weeks, and resulted in symptomatic limb ischemia [20]. If a subject had experienced > 1 endpoint, the first event to occur was used to define the onset of disease. The occurrence of the study outcomes was adjudicated on July 31, 2018, and it was based on a review of all of the electronic medical records and death certificates by an ad hoc committee of clinicians.

All of the laboratory assays undertaken at baseline and during follow-up were conducted within the medical laboratory department at Tawam Hospital. The circulating HbA1c levels were measured using an automated analyzer (Integra 400 Plus; Roche Diagnostics GmbH, Mannheim, Germany). Fasting lipid profiles and serum creatinine levels were measured using standard methods and a UniCel DxC-800 Synchron Clinical System (Beckman Coulter, Inc., Brea, CA, USA).

### Statistical analyses

The data distributions and categories were examined, and the categories with small sample sizes and skewed distributions were documented. The continuous variables are presented as the means (standard deviations) or as the medians (1st quartile, 3rd quartile), and the categorical variables are presented as proportions. Differences between men and women at baseline were assessed using the independent samples t-test if the data for the continuous variables were normally distributed and the Mann-Whitney U test if the data for the continuous variables were non-normally distributed, and Fisher’s exact test (2-tailed) was used for the categorical variables.

The patient-years at risk for major CVD, MI, stroke, and acute peripheral arterial occlusion were calculated for each subject from the baseline visit to the time at which each outcome was diagnosed, the time of death, or the time of the last outpatient clinic visit, whichever occurred first. The incidence rates and 95% confidence intervals (CIs) were calculated by dividing the number of new events for each outcome by the respective patient-years at risk. Kaplan-Meier time-to-event analyses were conducted for men and women for major CVD, MI, stroke, and acute peripheral arterial occlusion, and the results were compared using log-rank tests.

Multivariable Cox proportional hazards regression analyses stratified according to sex were used to determine the predictors of incident CVD. Since previous studies’ findings have shown that the SBP, DBP, HbA1c level and BMI have curvilinear associations with the risk of incident CVD [21, 22], quadratic terms for these predictors were initially considered in the models and they were excluded if they were not statistically significant. The proportional hazards assumption was assessed using log-log plots. Multicollinearity was evaluated by examining the tolerance. The results are expressed as the hazard ratios (HRs) and 95% CIs.

A value of *P* < 0.05 was considered statistically significant. All of the statistical analyses were performed using IBM®SPSS® software, version 25 (IBM Corporation, Armonk, NY, USA).

## Results

### Baseline characteristics

Table 1 shows the cohort’s baseline characteristics and a comparison of men and women. Compared with men, women were older and took lipid-lowering medication more frequently, and they had a higher BMI, higher TC and HDL-C levels, a higher eGFR, a lower TG level, a lower TC/HDL-C ratio, lower SBP and DBP, and were less likely to have a history of smoking. Among patients with DM, women had better glycemic control than men.

**Table 1:** Comparison of the patients’ baseline characteristics according to sex

Characteristic	Total (*n* = 977)	Men (*n* = 485)	Women (*n* = 492)	*P*-value^a^
Age (years)	49.72 (15.05)	48.00 (16.54)	51.40 (13.22)	< 0.001
Age (years), n (%)				
≤ 34	211 (21.6)	138 (28.5)	73 (14.8)	< 0.001
35–44	146 (14.9)	75 (15.5)	71 (14.4)	
45–54	227 (23.2)	84 (17.3)	143 (29.1)	
55–64	216 (22.1)	92 (19.0)	124 (25.2)	
≥ 65	177 (18.1)	96 (19.8)	81 (16.5)	
Males, *n* (%)	485 (49.6)	–	–	–
Smoking history, *n* (%)	171 (17.5)	165 (34.0)	6 (1.2)	< 0.001
Obesity, *n* (%)	462 (47.3)	184 (37.9)	278 (56.5)	< 0.001
DM, n (%)	418 (42.8)	208 (42.9)	210 (42.7)	1.00
Glycemic control^b^, n (%)				
HbA1c < 7.0%	189 (45.2)	76 (36.5)	113 (53.8)	< 0.001
HbA1c ≥ 7.0%	229 (54.8)	132 (63.5)	97 (46.2)	
HTN, *n* (%)	598 (61.2)	302 (62.3)	296 (60.2)	0.512
HTN group, *n* (%)				
Normal	643 (65.8)	313 (64.5)	330 (67.1)	0.441
Stage 1 HTN, *n* (%)	254 (26.0)	127 (26.2)	127 (25.8)	
Stage 2 HTN, *n* (%)	80 (8.2)	45 (9.3)	35 (7.1)	
Dyslipidemia, *n* (%)	854 (87.4)	440 (90.7)	414 (84.1)	0.002
CKD stages 3–5, *n* (%)	64 (6.6)	38 (7.8)	26 (5.3)	0.121
Treatment				
Antidiabetic drugs, *n* (%)	344 (35.2)	175 (36.1)	169 (34.3)	0.592
Antihypertensive agents, *n* (%)	523 (53.5)	253 (52.2)	270 (54.9)	0.405
Lipid-lowering medication, *n* (%)	481 (49.2)	217 (44.7)	264 (53.7)	0.006
Anthropometric parameters				
BMI (kg/m^2^)	29.65 (26.21, 33.58)	28.40 (25.21, 32.01)	30.97 (27.10, 35.68)	< 0.001
SBP (mmHg)	131.59 (17.94)	133.22 (17.17)	129.97 (18.53)	0.005
DBP (mmHg)	77.75 (11.35)	79.31 (11.73)	76.22 (10.76)	< 0.001
Laboratory parameters				
TC (mmol/L)	5.03 (1.11)	4.94 (1.13)	5.12 (1.09)	0.008
HDL-C (mmol/L)	1.09 (0.90, 1.33)	0.99 (0.84, 1.19)	1.19 (1.00, 1.43)	< 0.001
LDL-C (mmol/L)	3.28 (0.97)	3.25 (0.99)	3.31 (0.95)	0.271
TG (mmol/L)	1.11 (0.78, 1.64)	1.19 (0.82, 1.75)	1.05 (0.73, 1.50)	< 0.001
TC/HDL-C ratio	4.70 (1.39)	5.05 (1.41)	4.35 (1.28)	< 0.001
eGFR (mL/min.1.73 m^2^)	98.76 (22.89)	96.25 (24.56)	101.23 (20.85)	0.001
HbA1c (%)	6.00 (5.50, 6.90)	6.00 (5.50, 7.10)	5.91 (5.50, 6.75)	0.548

*CKD* chronic kidney disease, *DM* diabetes mellitus, *HTN* hypertension, *BMI* body mass index, *eGFR* estimated glomerular filtration rate, *SBP* systolic blood pressure, *DBP* diastolic blood pressure, *TC* total cholesterol, *TG* triglyceride, *HDL-C* high-density lipoprotein-cholesterol, *LDL-C* low-density lipoprotein-cholesterol, *HbA1c* glycosylated hemoglobin A1c

The data presented are the means and standard deviations, proportions, or medians (1st, 3rd quartiles)

^a^The independent-samples t-test was used to calculate the *P*-values for the continuous variables, and Fisher’s exact test (2-tailed) was used to calculate the *P*-values for the categorical variables. The Mann-Whitney U test was used to compare the median values of the HDL, TG, and HbA1c levels, and the BMI

^b^Glycemic control in patients with DM, total (*n* = 418), men (*n* = 208), and women (*n* = 210)

### Incidence of major cardiovascular events

During the median follow-up period of 8.92 years (interquartile range 7.85, 9.58 years), 97 subjects experienced incident major CVD, 66 subjects experienced incident MIs, 31 subjects experienced incident strokes, and 4 subjects experienced incident acute peripheral arterial occlusion. Overall, the incidence rate (per 1000 person-years) for major CVD was 12.7 (95% CI 10.4–15.4), and in men and women the incidence rates (per 1000 person-years) for major CVD were 16.8 (95% CI 12.9–21.4) and 9.0 (95% CI 6.4–12.4), respectively. The 9-year cumulative incidence of major CVD was 9.9% (95% CI 8.2–11.9). Major CVD and MI occurred significantly more frequently in men than in women, and the rates of stroke and acute peripheral arterial occlusion were similar in men and women (Table 2).

**Table 2:** Nine-year cumulative incidence and incidence rates of cardiovascular disease in high-risk nationals from the United Arab Emirates

		*n*	Total (*n* = 977)	*n*	Men (*n* = 485)	*n*	Women (*n* = 492)	*P-*value^a^
Major CVD	Cumulative incidence^b^	97	9.9 (8.2–11.9)	61	12.6 (9.9–15.8)	36	7.3 (5.3–9.9)	0.003
	Incidence rate^c^		12.7 (10.4–15.4)		16.8 (12.9–21.4)		9.0 (6.4–12.4)	
MI	Cumulative incidence^b^	66	6.8 (5.3–8.5)	43	8.9 (6.6–11.7)	23	4.7 (3.1–6.8)	0.005
	Incidence rate^c^		8.5 (6.6–10.8)		11.6 (8.5–15.5)		5.7 (3.7–8.4)	
Stroke	Cumulative incidence^b^	31	3.2 (2.2–4.4)	17	3.5 (2.1–5.4)	14	2.9 (1.6–4.6)	0.452
	Incidence rate^c^		4.0 (2.7–5.5)		4.5 (2.7–7.1)		3.4 (2.0–5.6)	
Acute peripheral arterial occlusion	Cumulative incidence^b^	4	0.4 (0.1–1.0)	3	0.6 (0.2–1.7)	1	0.2 (0.0–1.0)	0.266
	Incidence rate^c^		0.5 (0.2–1.2)		0.8 (0.2–2.1)		0.2 (0.0–1.2)	

*CVD* cardiovascular disease, *MI* myocardial infarction

^a^The log-rank test was used to calculate the *P*-values

^b^The data presented are the cumulative incidence percentages (95% confidence intervals)

^c^The data presented are the incidence rates (cases/1000 person-years) (95% confidence intervals)

### Analyses of risk factors

Table 3 presents the results from the multivariable Cox regression analyses of incident major CVD. The tolerance ranged from 0.45 to 0.98, indicating an absence of multicollinearity, and the log-log plot to test the proportional hazards assumption was not significant. In both sexes, a low eGFR was a significant risk factor for major CVD. The SBP was a strong predictor of major CVD, with risk increases of 20 and 58% per 10 mmHg SBP increase in men and women, respectively. The major CVD risk increased by 16% in men and 24% in women for each 1% rise in the HbA1c level. In women, the TC/HDL-C ratio was an additional significant independent risk factor, with an estimated 44% risk increase per ratio increment. Age and a history of smoking exacerbated the major CVD risk by 50% per decade and 80%, respectively, in men. In the model, the quadratic terms of the BMI, SBP, DBP, and HbA1c level were not statistically significant in either men or women.

**Table 3:** Adjusted^a^ hazards ratios and 95% confidence intervals for the predictors of cardiovascular disease

Predictor variable	Men (*n* = 485)		Women (*n* = 492)	
	HR (95% CI)	*P-*value	HR (95% CI)	*P-*value
Sociodemographic variables				
Age per 10 years	1.50 (1.19–1.89)	0.001	1.10 (0.77–1.56)	0.597
History of smoking	1.80 (1.07–3.02)	0.027	0.00 (0.00–3.07)	0.967
Treatment modalities				
Antidiabetic drugs	0.87 (0.41–1.82)	0.706	1.67 (0.67–4.15)	0.274
Antihypertensive agents	0.84 (0.42–1.68)	0.621	0.73 (0.28–1.93)	0.530
Lipid-lowering medication	0.81 (0.44–1.49)	0.497	1.04 (0.42–2.58)	0.935
Anthropometric parameters				
BMI (kg/m^2^)	1.00 (0.96–1.05)	0.982	0.96 (0.92–1.02)	0.175
SBP per 10 mmHg	1.20 (1.01–1.44)	0.042	1.58 (1.27–1.96)	< 0.001
DBP per 10 mmHg	1.00 (0.77–1.31)	0.998	0.71 (0.50–1.01)	0.056
Laboratory parameters				
TC/HDL-C ratio	0.95 (0.77–1.17)	0.613	1.44 (1.02–2.04)	0.037
LDL-C (mmol/L)	1.10 (0.83–1.44)	0.516	0.80 (0.50–1.30)	0.365
eGFR (mL/min.1.73 m^2^)	0.98 (0.97–0.99)	< 0.001	0.98 (0.97–1.00)	0.043
HbA1c (%)	1.16 (1.02–1.32)	0.029	1.24 (1.03–1.49)	0.022

*BMI* body mass index, *eGFR* estimated glomerular filtration rate, *SBP* systolic blood pressure, *DBP* diastolic blood pressure, *TC* total cholesterol, *HDL-C* high-density lipoprotein-cholesterol, *LDL-C* low-density lipoprotein-cholesterol, *HbA1c* glycosylated hemoglobin A1c, *HR* hazards ratio, *CI* confidence interval

^a^Multivariable Cox model adjusted for all of the predictors in the final model

## Discussion

To the best of our knowledge, this is the first long-term study that has investigated the incidence rates and potential predictors of CVD among high-risk Emirati men and women. The incidence rate of major CVD per 1000 person-years was 12.7, and the 9-year cumulative incidence of major CVD was 9.9%. When stratified according to sex, the TC/HDL-C ratio in women and age and smoking history in men were significant predictors of incident major CVD. The SBP, eGFR, and HbA1c level were risk factors that were strongly associated with major CVD in both sexes.

Globally, the incidence rates of CVD vary among different high-risk populations. The findings from a recent 5-year study conducted in a neighboring Arab country showed that the incidence rate of CVD among patients with diabetes was 17.6 per 1000 person-years, which is higher than the incidence rate determined in this study [6]. A Chinese study’s findings showed a CVD incidence rate of 17.2 per 1000 person-years among high-risk patients over 5 years [21]. The findings from a study of Italian patients with DM reported higher CVD incidence rates of 28.8 and 23.3 per 1000 person-years among men and women, respectively [23]; however, this study’s outcome was coronary heart disease only, and its follow-up period was just 4 years. In contrast, an 11-year population-based study of patients with DM in India revealed a CVD incidence rate of 5.6 cases per 1000 person-years [24]. Methodological differences, including the inclusion criteria, incident CVD definitions, and the follow-up durations, may have caused the different CVD incidence rates; therefore, direct comparisons of the studies’ findings may not be feasible.

Age is a major non-modifiable risk factor that is associated with incident major CVD [25, 26]. After adjusting for other risk factors, age was found to be a significant predictor of major CVD among men, but not among women in our study. The average age of patients with CVD in Europe and in North America is 60 to 65 years [27], as compared with 56 years in a multi-center middle eastern population-based study [28]. The mean age of 50 years in our study population suggests a younger population at risk of CVD. This stresses the need for early recognition of CVD and its risk factors in the UAE population.

In our study, we found that the incidence rate of major CVD was higher in men than in women. Compared with women, incident MI was twice as frequent in men, but the sexes did not differ in relation to incident stroke and acute peripheral arterial occlusion. The sex-specific differences in incident CVD observed in our study may have been caused by the effects of unconventional risk factors in women, including pregnancy-related disorders, the menopausal status, and hormone and other therapies [29–32].

Our results showed that compared with non-smokers, men with a history of smoking had a significantly higher risk of CVD. Cigarette smoking negatively influences other traditional cardiovascular risk factors, for example, DM and serum lipids [33–35], and it has a multiplicative association with HTN on incident CVD [36]. Our study’s findings indicate that effective smoking cessation programs must be implemented by healthcare authorities at a national level to prevent CVD.

Intriguingly, our study’s results showed that while women’s baseline TC/HDL-C ratio was lower (*P* < 0.001) than that in men and they used lipid-lowering medications more frequently (*P* < 0.006) than men, a high TC/HDL-C ratio was significantly associated with CVD risk in women, but not in men. We are unable to deduce the reason(s) underlying this sex-specific phenomenon; however, in women, the TC/HDL-C ratio as a biomarker is more strongly correlated with CVD than LDL-C alone [37]. Further studies are necessary to determine the role of lipoproteins in CVD risk-stratification in this population.

CKD is a risk factor for incident CVD [38, 39]; consequently, the current clinical guidelines classify patients with CKD as “high-risk” for CVD [18, 40]. Our study’s results showed that a low eGFR was a significant risk factor for incident major CVD in both men and women. Therefore, local health authorities should promote screening for CKD in clinical practice to facilitate predictions of CVD in high-risk patients.

The HbA1c level, which reflects glucose control among patients with DM, was a significant predictor of incident CVD in both sexes in this study, a finding that concurs with the results from previous studies that suggest the CVD risk increases in patients as their HbA1c levels increase [41–43]. In addition, a significant linear association between the HbA1c level and incident CVD was observed in our study. However, contrary to this finding, other studies’ findings have demonstrated curvilinear relationships between the HbA1c level and incident CVD [21, 44, 45]. Our results suggest that future public health campaigns that target CVD and DM should focus on lifestyle and other related risk factors among high-risk individuals.

We found that a higher SBP strongly predicted incident CVD in both sexes, which concurs with the findings from other longitudinal studies [21, 46]. Curvilinear relationships between the DBP, SBP, and CVD have been reported from observational studies [45, 47], but these were not evident in our study. Despite the recognition of the importance of the SBP for reducing the CVD risk and the emphasis on SBP control in recently updated guidelines [48], a considerable number of patients fail to achieve SBP targets [49, 50]. The findings from a regional survey of the prevalence, awareness, treatment, and control of HTN in 4 countries in the Middle East, including the UAE, showed that approximately 33% of the participants had HTN, of whom 47% were being treated with antihypertensive medications, and, of these treated subjects, only 19% had BPs < 140/90 mmHg [50]. Thus, effective strategies for the management of HTN must be implemented to control the SBP and minimize the CVD risk. For example, specialized clinics that are involved in HTN treatment could be established that emphasize the long-term value of treatment compliance through patient education.

### Strengths and limitations

One strength of this study is that it is the first longitudinal study to assess the epidemiology of CVD and sex-specific risk factors in high-risk UAE nationals with a median follow-up period of 9 years. A longer follow-up period is desirable as this would enable the long-term risk factors associated with CVD to be determined. Moreover, this study’s analyses relied on direct measurements of the laboratory variables and anthropometric parameters rather than self-reported information to classify the predictor variables.

This study has several limitations. First, this was a retrospective study. A prospective study design involving standardized measurements of the laboratory variables and anthropometric parameters may have furnished the investigation with data of better quality and reduced the potential for bias. Second, other parameters, for example, the waist circumferences, family histories of CVD, physical activity, diet, and the menopausal status were not included in this study, because the data were unavailable, and could have impacted the study outcomes. Third, our study consisted of UAE nationals at high cardiovascular risk from outpatient clinics of a single hospital, therefore, our results may not be applied to the general population in the UAE. Finally, changes in the status of medication use and the evolution of risk factors from baseline were not considered during the follow-up period. These and other confounding factors affecting the study outcomes cannot be ruled out. The changes in risk factors over time and their impact on CVD outcome may be worth assessing in future studies.

## Conclusions

In conclusion, men had a greater risk of incident major CVD than women during this study’s 9-year follow-up period. A high SBP, low eGFR, and an elevated HbA1c level were independent risk factors associated with major CVD in both sexes. Age and a history of smoking among men and a high TC/HDL-C ratio in women were additional risk factors associated with incident major CVD. These results indicate that sex-specific risk factors must be considered in relation to the primary prevention of incident CVD among high-risk UAE nationals. Lowering the TC/HDL-C ratio in conjunction with LDL-C level reductions should be considered, particularly for Emirati women with dyslipidemia.

## Additional file

Additional file 1: Dataset. Incidence of cardiovascular disease and associated risk factors in at-risk men and women in the United Arab Emirates: a 9-year retrospective cohort study. *ID* identification, *DM* diabetes mellitus, *HTN* hypertension, *HDL-C* high-density lipoprotein cholesterol, *LDL-C* low-density lipoprotein cholesterol, *HbA1c* glycosylated hemoglobin, *eGFR* estimated glomerular filtration rate, *SBP* systolic blood pressure, *DBP* diastolic blood pressure, *BMI* body mass index, *CVD* cardiovascular disease. (XLSX 126 kb)

## Data Availability

The dataset supporting the conclusions of this article is included within the article and its Additional file 1.

## Abbreviations

BMI: Body mass index
BP: Blood pressure
CI: Confidence interval
CKD: Chronic kidney disease
CVD: Cardiovascular disease
DBP: Diastolic blood pressure
DM: Diabetes mellitus
eGFR: Estimated glomerular filtration rate
HbA1c: Glycosylated hemoglobin A1c
HDL-C: High-density lipoprotein-cholesterol
HR: Hazards ratio
HTN: Hypertension
LDL-C: Low-density lipoprotein-cholesterol
MI: Myocardial infarction
SBP: Systolic blood pressure
TC: Total cholesterol
TG: Triglycerides
UAE: United Arab Emirates
